# Plastic Filling

**Published:** 1876-12

**Authors:** S. P. Cutler

**Affiliations:** Memphis, Tennessee.


### AMERICAN JOURNAL

OF

### DENTAL SCIENCE.

**Vol. X. THIRD SERIES—DECEMBER, 1876. No. 8.**

### ARTICLE I.

### *Tennessee Dental Association.—Report of Committee on Plastic Filling.*

BY S. P. CUTLER, M. D., D. D. S., MEMPHIS, TENNESSEE.

The subject of plastic fillings necessarily embraces all that class of materials that are plastered into the cavity of decay, in a soft or plastic state or condition, but which harden in the tooth afterwards. Under this head we have quite a number, equally as numerous as all other materials combined. In this report amalgam claims the largest share of the discussion, it being the only one that can be called permanent or durable in its character.

Gutta-percha, in its various combinations, or as simple gum, is valuable whenever indicated, as a temporary stopping. For a stopping Hill's preparation is the most durable, and when thoroughly introduced in some mouths will last several years. In frail shells of teeth it is peculiarly valuable, in fact it has been too much supplanted by *os-artificial* of late

The gum dissolved in chloroform is one of the best preparations we have for filling nerve cavities and fangs, when admissible, the chloroform evaporating immediately leaves the gum behind, filling every point; in lower teeth it may be dropped in, in upper teeth it will have to be forced up with probes and pledges of cotton.

In this form it is sometimes used to great advantage, as a capping over exposed, or nearly exposed nerves, with gold cap over it, or not, as the case may be; it is a non-irritant and non-conductor, the gum alone may be used for this purpose by applying heat. I have used our common shelac varnish sometimes for fang filling with success, also the gum as temporary stoppings by using heated instruments hydrate of the oxychloride of zinc, or os-artificial, bone filling, Guillois' cement, cement plombe, &c.

These preparations are all about the same thing chemically, and are invaluable adjuncts in the hands of the dentist, and too much praise cannot be bestowed upon the man first introducing them; the profession now could hardly get along without them; for one I could not think of it. Bone filling is our man of all work, our *aneella* in the profession; we might ask what do we use it for? In reply; sayeth the yankee, "what *dont* we use it for verily?" As a pain obtunder in sensitive teeth it is our *sine quanon.*

It has been, and will continue to be the cause of saving thousands of teeth, that otherwise would be lost, be suffered to decay out rather than suffer the pain of filling—in fact the merits of this article are too little understood by a great many in the profession.

How does this material produce obtundency? Let us investigate. Hyporæsthesia or exalted sensibility, is often met within almost a majority of cases, governed greatly by temperament. Why this is so I will not now argue.

The white variety of decay is oftener sensitive than any other as a rule, and most destructive in its ravages. In this variety chemical action of acids is most active, rapidly penetrating and disintegrating the phosphate of lime, leaving more fibrils extensively exposed to this chemical action within the disintegrating substance; the fibrils still retaining vitality are kept fired or kindled up to a high tension. In the black variety of decay, the process is the slowest of any, the action of the acids not penetrating scarcely below the surface of decay, hence there is not the same extent of softened dentine embracing fibrilan extremities of nerves, in consequence not so much sensibility.

In the brown or intermediate variety, the action of the acids completely dissolve out the triple phosphate of lime down some distance, in a slow manner, leaving behind the animal portion or cartillage with dead fibrils within this decayed mass, which mass may often be lifted out from almost sound dentine below, leaving a smooth surface; this is the dead line in this case. This tough mass protects the fibrils below from external influences to some extent. The miscroscope shows the tubular structure in this matrix to be almost perfect; some of them are seen to be enlarged, but seldom any fungi or parasites are seen in this mass. It is in the white variety of decay that the bone filling is most indicated as an obtunder, the hydrochloric acid in the preparation, when first introduced, that acts on the sensitive nerve extremities, as a caustic, then exciting a slight astringent effect for a time, then as a protection to the decayed dentine from external influences, it thus arrests further decay. It may be put in at first without any cleaning out of cavity if it is very sensitive, only syringing well with warm water. It may be necessary to repeat the application several times, cleaning out a little each subsequent time if necessary; days, weeks and months may be necessary to accomplish the object, varying in different cases. Often times at first, medicating with creasote or other agents may be necessary in order to prevent pain when the pulp is near, from the caustic action.

As a nerve ossifier there is no remedy equal to it. As a capping under permanent fillings, it is one of the best, when indicated.

For filling nerve cavities and fangs, it is equal if not preferable to gutta-percha or anything else in my estimation.

For temporary fillings for any purpose, and permanent filling for very frail shells of teeth, when too weak to hold gold, tin or amalgam, it serves a valuable purpose so long as it lasts. Experience gives success; much more might be said on the subject. Other plastic materials, for various purposes are sometimes used for fang fillings, and temporary stopping, as Stearine, Wax, Parafine, Gypsum, and with success.

Next we will notice, fusible alloy as a permanent stopping more for temporary purposes; this when properly prepared and properly introduced, in many instances, serves a good purpose, though not used in this country now; when molars need building out, or when the walls are frail, it is equal to amalgam; it holds its color better than amalgam, and never disintegrates around the edges, as is sometimes witnessed in the case of amalgam fillings.

It may be melted in the palm of the hand without burning the skin.

In making the alloy only pure tin and bismuth should be used; a trifle of mercury makes it melt at a lower heat, though this is not necessary. This filling should be softened over a spirit lamp, then with a heated plugger suited to the purpose, burnished into the cavity; the cavity if large may not be filled full at first, bnt the bottom may be first filled and well burnished all around until cold, then fill up and continue to burnish and condense thoroughly until cold, otherwise some shrinkage may take place This filling is harder than block tin, and will not wear to any extent; it may be put into frail front teeth successfully. Some teeth will not bear the heat well; in dead teeth there is no difficulty; it is a peer by the side of amalgam, without any of the objections of the latter; when finished up well, it is a thing of beauty to look at. In Germany many years ago, 30 or 40 years, it was extensively used, though the material employed was dark and unsightly. It has never been used much in this country, only by German dentists years gone by. This compound should come into general notice.

Last but not least, comes in our old friend amalgam, for a share of praise as a plastic filling material. I am well acquainted with all the objections that have been raised against its use, and accept them for what they are worth. As to any poisonous effects following the use of this article from the presence of mercury, I must say in candor, that I have the first case to see yet; some others talk differently.

Some contend that this material tends to destroy pulps; this has not been the result of my own observation; on the contrary, it probably tends to preserve pulps and harden the dentine by forcing tubular ossification just under the filling; the dark oxide sometimes found under the filling, is argentic oxide, and not black oxide of mercury, as the conditions do not there exist to form this oxide, but do exist for the formation of silver oxide to a limited extent. From the fact that we often find the cavity under an amalgam filling harder than ordinary dentine, more like enamel, we may infer that this effect has been brought about by the presence of this oxide, the nature of which change is already explained above.

From microscopic observations I have arrived at the above conclusions; though we need much more microscopic light on this subject. If such injurious consequences as have been alleged, follow the use of this material, communities, as well as the dentists, would find it out, as it has been extensively used, and condemn it broad cast; this has not been done, even with the influence of the opponents in the profession, brought to bear, as it has been, in communities, against its use.

We find in some cases, when a tooth is filled with this compound, that if any metalic body, as clasp of a plate or knife blade, touches it, a painful shock is felt, though otherwise comfortable; this is objectionable. If this is not remedied by time the filling should be drilled out and the cavity treated with os-artificial, then refilled. Such teeth do not undergo calcification readily under the filling, judging from some few observations made; this condition ismost likely to occur in cases of white decay, when there has been no previous treatment.

As a filling for fangs I would not recommend amalgam, from the fact that it could not be as readily forced into minute canals as some other materials mentioned above, and there might be some danger of the vapors of the mercury escaping through the foramen of the fang into the alveolus and out through the gums, causing some mechanical disturbance and irritation.

Now as to the many disastrous failures we hear and read about; they no doubt mainly depend upon the following facts namely—want of proper knowledge concerning its properties, physical, chemical and metalurgical, which are not well understood by many in the profession who use the material.

By many it is used as a last resort only in preference to the forceps; of course this material is preferable to the forceps. The careless manner in its use in general, is another draw back to its reputation ; a want of thoroughness in all detail comes back on the operator, and the thing is condemned in disgust. It is often stuffed into cavities half cleaned out, not dryed, not condensed properly, not finished off properly; into dead teeth without first filling the fangs with something else, on exposed nerves, into teeth diseased in alveolus, without any previous treatment at all ; often times it is left projecting beyond border of cavity at gums, causing irritation and inflammation. In filling teeth with amalgam, just the same care and thoroughness in every particular, should be used as in the case of gold, then success may be looked for.

In filling a tooth with a large cavity, fill about half full at first, then thoroughly condense with a square end instrument, then burnish all around with a round headed burnisher, then fill full, or if extra large, make as many as 3 or 4 separate applications, proceeding each time in the same way, and finish up on some subsequent day, never on the same day.

Crowns of teeth may readily be built out with this material. In front upper fangs with extensive funnel shaped hollows, where a pivot tooth is needed the root may be cleaned out thoroughly and filled, either around a silver cylinder or a steel pin slightly tapering, which last may be left in until next day, then removed with a small pair pliers by rotating the pin gently, and then drilling them to suit pivot; in this way Ihave made bad looking fangs usefull for many years.

As a filling for deciduous teeth I often use amalgam, never gold, as it is not so good. Tedious operations often make a lasting unfavorable impression, causing future dread in dental operations ; we should think of this. A lady friend of mine told me the other day that I filled upper molars on each side with amalgam for her about 30 years ago. Some 4 years ago she had them pulled, the last in the upper jaw, the fillings were in their places sound and firm, said she done most of her chewing on them for years, she says. I told her at the time that they were not worth plugging and would last only a few years; the dentist who extracted them waa surprised. The material was coin silver and mercury, old style and dark. There is a great difference in quality of amalgams in the market, though more depends on manipulation than in quality. Clear white looking is the best, ashy or dirty looking is not so good, and appear discolored when finished ; some retain a bright color, others do not; some discoler the dentine more than others. The prices charged for most amalgams are out of all reason, $2.00 pays well. The zinc preparations pay heavy profits, they are too high. Some amalgams require more mercury than others, such as contain most silver, and are the hardest, though perhaps no better ; some can be worked dryer than others; In some cases amalgam may be used very dry in others not so much so ; the rule varies to suit circumstances, experience being the best guide.

